# Brexpiprazole: A new option in treating agitation in Alzheimer's dementia—Insights from transgenic mouse models

**DOI:** 10.1002/npr2.12461

**Published:** 2024-06-25

**Authors:** Naoki Amada, Shinji Sato, Dai Ishikawa, Mai Nakamura, Mikio Suzuki, Takashi Futamura, Kenji Maeda

**Affiliations:** ^1^ Otsuka Pharmaceutical Co., Ltd. Tokushima Japan; ^2^ Otsuka Pharmaceutical Co., Ltd. Tokyo Japan

**Keywords:** aggression, agitation, Alzheimer's disease, behavioral and psychological symptoms associated with dementia (BPSD), brexpiprazole

## Abstract

**Aim:**

Brexpiprazole is the first FDA‐approved treatment for agitation associated with dementia due to Alzheimer's disease. Agitation in Alzheimer's dementia (AAD) occurs in high prevalence and is a great burden for patients and caregivers. Efficacy, safety, and tolerability of brexpiprazole were demonstrated in the AAD clinical trials. To demonstrate the agitation‐ameliorating effect of brexpiprazole in animals, we evaluated brexpiprazole in two AAD mouse models.

**Methods:**

The resident–intruder test was conducted in 5‐ to 6‐month‐old Tg2576 mice, given vehicle or brexpiprazole (0.01 or 0.03 mg/kg) orally 1 h before the test. Locomotor activity was measured in 6‐month‐old APPSL‐Tg mice given vehicle or brexpiprazole (0.01 or 0.03 mg/kg) orally the evening before the start of locomotor measurement for 3 days.

**Results:**

In the resident–intruder test, Tg2576 mice showed significantly higher attack number and shorter latency to first attack compared to non‐Tg mice. In the Tg mice, brexpiprazole treatment (0.03 mg/kg) significantly delayed the latency to first attack and showed a trend toward a decrease in attack number. APPSL‐Tg mice (≧6 months old) showed significantly higher locomotion during dark period Phase II (Zeitgeber time [ZT] 16–20) and Phase III (ZT20‐24) compared to non‐Tg mice, correlating with the clinical observations of late afternoon agitation in Alzheimer's disease. Brexpiprazole treatment (0.01 and 0.03 mg/kg) significantly decreased hyperlocomotion during the Phase III in APPSL‐Tg mice.

**Conclusion:**

The suppression of attack behavior and the reduction of nocturnal hyperlocomotion in these Tg mice may be indicative of the therapeutic effect of brexpiprazole on AAD, as demonstrated in the clinical trials.

## Abstract 2

Brexpiprazole was evaluated on agitation‐related behaviors in two transgenic mouse models of Alzheimer's dementia. Brexpiprazole suppressed attack behavior and reduced nocturnal hyperlocomotion in these model mice, which may be indicative of the therapeutic effect of brexpiprazole on agitation in Alzheimer's dementia, as demonstrated in the clinical trials.

## Graphical abstract

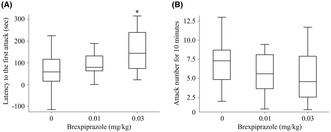

## 1 INTRODUCTION

Alzheimer's dementia is a chronic neurodegenerative condition characterized by the progressive deterioration of cognitive function and memory as well as behavioral and psychological symptoms, which is a growing global health and economic issue as elderly populations increase dramatically across the world.^1^, ^2^, ^3^ The importance of behavioral and psychological symptoms associated with dementia has been mentioned since the concept of dementia was first proposed, and it is noted that Auguste Deter, who was first diagnosed with Alzheimer's dementia, had behavioral symptoms such as jealous delusions, hallucinations, agitation, insomnia, and screaming, in addition to core symptoms such as memory and disorientation problems.^4^, ^5^ In 1996, the concept of “behavioral and psychological signs and symptoms of dementia” was proposed by Finkel et al.^6^ at the International Psychogeriatric Association. Based on this, the consensus conference in 1999 defined the term “behavioral and psychological symptoms of dementia (BPSD)” to describe the cognitive, thought content, mood, and behavioral disturbances often seen in patients with dementia. BPSD causes high levels of distress to Alzheimer's dementia patients and their caregivers, and is also associated with poor outcomes and increased use of medical resources. Therefore, in addition to cognitive decline, BPSD is a clinical issue to be thrustfully intervened.

A recent study reported that the prevalence of at least one BPSD in patients with Alzheimer's dementia was high (90.8%).^7^ The most frequent symptoms in patients with Alzheimer's dementia were apathy (57.4%), irritability/affective lability (50.5%), agitation/aggression (42.3%), and sleep/nighttime behavior (35.6%).^7^ Given these high frequency and impact on management and overall healthcare resources, BPSD should be considered an important diagnostic and treatment target in Alzheimer's dementia.

Brexpiprazole is a serotonin–dopamine activity modulator that acts as a partial agonist at serotonin 5‐HT_1A_ and dopamine D_2_ receptors, and as an antagonist at serotonin 5‐HT_2A_ and noradrenaline α_1B_/α_2C_ receptors, all at pharmacologically relevant potency.^8^ In 2015, the FDA‐approved brexpiprazole as a treatment for schizophrenia and as an adjunctive therapy for major depressive disorder (MDD).^9^ Brexpiprazole has a low propensity for activating and sedating side effects.^10^ Given Otsuka's research experience with brexpiprazole, it was hypothesized that brexpiprazole may provide a benefit in the management of Alzheimer's dementia patients who exhibit agitation. From this perspective, in order to investigate the efficacy, safety, and tolerability of brexpiprazole in patients with agitation in Alzheimer's dementia (AAD), three randomized, double‐blind, placebo‐controlled Phase III trials have been carried out.^11^, ^12^ These studies indicated evidence of brexpiprazole to be an efficacious, safe, and well‐tolerated treatment for AAD.^12^ In 2023, based on these clinical trial data, the FDA approved an additional indication of brexpiprazole for the treatment of AAD.

Increased hostility and aggression are part of the BPSD seen in Alzheimer's dementia. In general, amyloidgenic and tauogenic model mice exhibit increased aggression relative to wild‐type controls.^13^ The resident–intruder test is considered the standard for assessing aggression in mice^14^ and has been successfully used to assess aggressive behavior in a number of mouse strains. In this behavioral paradigm, after a period of socially isolated rearing, a novel intruder is introduced into the home cage of the subject (resident), usually resulting in attack behavior such as biting and kicking by the resident on the intruder. Alexander et al.^15^ reported a BPSD model focusing on aggression using Tg2576 mice, one of the most versatile Alzheimer's dementia model mice in the world, which has Swedish and London‐type amyloid precursor protein (APP) mutations.^16^

In addition to the aggressive problems described above, patients with Alzheimer's dementia also show circadian rhythm disturbances, such as disrupted sleep–wake cycles, nocturnal hyperactivity, and phase shifts in body temperature,^17^, ^18^, ^19^ Interestingly, in patients with dementia, agitation and circadian rhythm disturbances (i.e., sleep–wake and body temperature) are closely related in patients with dementia,^20^ and disruption of hypothalamic function may underlie their co‐occurrence.^21^ In Alzheimer's dementia mice model, Vloeberghs et al.^22^ reported spontaneous locomotor changes based on nocturnal circadian rhythms for 3 days in APP23 mice, which has a Swedish‐type APP mutation (KM670/671NL). Twelve‐month‐old APP23 mice showed a significant increase in nocturnal spontaneous locomotor activity during the second half of dark (active) period on Days 2 and 3 after starting the measurement than wild‐type mice, which was referred as reminiscent of sundowning syndrome, exacerbation of agitation in the late afternoon and early evening, in patients with Alzheimer's dementia.

In the present study, the pharmacological effects of brexpiprazole on BPSD‐like symptoms in Alzheimer's dementia models were evaluated, using resident intruder model in Tg2576 mice and the circadian rhythm alterations model in APPSL‐Tg mice^23^, ^24^ overexpressing a mutant form of APP with the Swedish and London mutations.

## 2 MATERIALS AND METHODS

### 2.1 Drug

Brexpiprazole was synthesized at Otsuka Pharmaceutical Co., Ltd. (Tokushima, Japan), and suspended in 5% (w/v) gum Arabic distilled water solution using agate mortar and diluted with the same solution.

### 2.2 Animals

For the resident–intruder tests, male Tg2576 mice [B6;SJL‐Tg(APPSWE)2576Kha] carrying the Swedish‐type APP mutation (K670N, M671L) and non‐Tg male mice were purchased from Taconic (New York, NY, USA), and reared and aged to 5–6 months. Male A/J mice were purchased from Japan SLC, Inc. (Shizuoka, Japan), as intruders.

For the circadian rhythm locomotor activity, male APP transgenic mice (APPSL‐Tg) and non‐Tg mice were used.^23^ This transgenic mouse line was established at Otsuka Pharmaceutical Co., Ltd.

### 2.3 Resident–intruder test

The Tg2576 mouse resident–intruder test has been conducted as described in detail before.^15^ Briefly, resident mice were housed in an experimental room maintained on a reversed 12:12 light/dark cycle. The resident mice were individually housed for 2 weeks in a cage (14 × 14.1 × 23.1 cm) filled with pine wood shavings. During this period, and throughout all social encounters, the bedding in the resident home cages was not changed in order to maintain the olfactory characteristics of a territory. Measurements were made during the first phase of the dark period (4 h) when mice show the highest locomotor activity. Each mouse was exposed to an experimentally naive and body‐weight‐matched or slightly smaller A/J male intruder mouse during a 10‐min session. Biting was recorded as aggressive behavior, and the time required for the first biting and the total number of biting over 10 min were measured. It has been reported that A/J mice did not show any nonaggressive behavior and response to attacks with retaliation against individually bred resident Tg2576 mice.^25^ Therefore, mice of this strain are often used as intruders in resident–intruder tests. The room was illuminated with less than 10 lux of light to allow unobtrusive observation of mouse behavior. To avoid injury, the social interaction was interrupted if the fighting escalated to the point that the resident mouse persistently bit the intruder male for more than 10 s and the test was restarted. Prior to performing the resident intruder test, the aggressiveness of Tg2576 mice was confirmed by a preliminary experiment using the same protocol, and then 43 mice that showed clear aggressive behavior were used to evaluate the effect of brexpiprazole.

Forty‐eight Tg2576 mice and 10 non‐Tg mice were screened beforehand by the resident–intruder test, and the promotion of aggression in Tg2576 mice was confirmed by statistical analysis (see Statistical Analysis, Section 2.7).

### 2.4 Brexpiprazole treatment of aggressiveness in Tg2576 mice

Based on body weight and latency to first attack in the preliminary experiment, 43 Tg2576 mice were assigned to three groups (1:1:1). One hour before the test, either brexpiprazole (0.01 and 0.03 mg/kg) or vehicle was administered orally to each resident mouse at a volume of 10 mL/kg. The doses were determined as nonsedative in B6SJLF1 mice, a strain commonly used for Tg2576 mouse colony maintenance.

### 2.5 Circadian rhythm locomotor activity

Spontaneous circadian rhythm locomotor activity was measured using Supermex (Muromachi Kikai Co., Ltd., Tokyo, Japan). Prior to testing, mice were individually acclimated for 1 h in the plastic test cage (Mouse Hi space TPX, W15.5 × H14.8 × D24.5 cm, CLEA Japan, Inc., Tokyo, Japan) inside a soundproof room and their locomotor activity was measured for 62.5‐h periods (3 nights and 2 days) under free food and water‐drinking conditions. Measurements started at 6:30 p.m. In this apparatus, a passive infrared sensor detects the signals emitted by the mouse, and the number of transpositions is counted. The readings were accumulated every 60 min and automatically total counts were calculated using specialized software CompACT AMS (Muromachi Kikai Co., Ltd.). The nocturnal spontaneous locomotor activity for each night over 3 days was counted. Each nocturnal locomotor activity during the night was divided and counted into three phases, every 4 h (Phase I: 7:00 p.m.–11:00 p.m., Phase II: 11:00 p.m.–3:00 a.m., and Phase III: 3:00 a.m.–7:00 a.m.), according to previous literature.^26^

### 2.6 Brexpiprazole treatment of nocturnal spontaneous locomotor activity in APPSL‐Tg mice

Newly, APPSL‐Tg and non‐Tg mice at 6 months of age were assigned to groups based on body weight and spontaneous locomotor activity during the dark period from 7:00 p.m. to 7:00 a.m. at the day before starting the test as indices. For 3 days, brexpiprazole (0.01 and 0.03 mg/kg) or vehicle was administered orally to the mouse at a volume of 10 mL/kg at every evening between 5:00 p.m. and 6:30 p.m. After dosing, measurement of locomotor activity was rapidly started or continued. To analyze the differences among the groups, spontaneous locomotor activity counts during each night were calculated every 4 h in three phases.

### 2.7 Statistical analysis

All statistical analyses were performed using SAS software for Windows (SAS Institute Japan, Tokyo, Japan). The differences were considered significant, if the *p*‐value was less than 0.05.

#### 2.7.1 Resident intruder test

Results were expressed as the box‐and‐whisker plot. To confirm the enhanced aggression and agitation of the Tg2576 mouse, analysis was performed using a Wilcoxon rank‐sum test compared to the non‐Tg mouse.

In order to evaluate the inhibitory effect of brexpiprazole on the aggressive behavior, analysis of covariance (ANCOVA) with a covariate (baseline value of each resident mouse before treatment) was conducted on the latency to attack and the number of attacks.

#### 2.7.2 Circadian rhythm locomotor activity

The data were presented as mean ± SEM.

At first, as a preliminary test, the nocturnal locomotor activities in each night (Night 1 to 3) between APPSL‐Tg and non‐Tg mice were compared at ages of 4.5, 6, 8, 11, and 12 months using an unpaired *t*‐test. Then, each nocturnal phase locomotor activity (Phase I, II, or III) in each night was also compared between APPSL‐Tg and non‐Tg mice using an unpaired *t*‐test.

In order to evaluate the inhibitory effect of brexpiprazole on the nocturnal locomotor activity, significant increase in the nocturnal locomotor activities in each night in APPSL‐Tg mice against non‐Tg mice were confirmed at age of 6 months using an unpaired *t*‐test. Then, the effect of brexpiprazole on each nocturnal phase locomotor activity in each night was statistically analyzed, with factors, time and dose, by the mixed‐effect model for repeated measures (MMRM method) followed by Dunnett's test.

## 3 RESULTS

### 3.1 Confirmation of aggressiveness of Tg2576 mice by preliminary resident intruder

**FIGURE 1:**
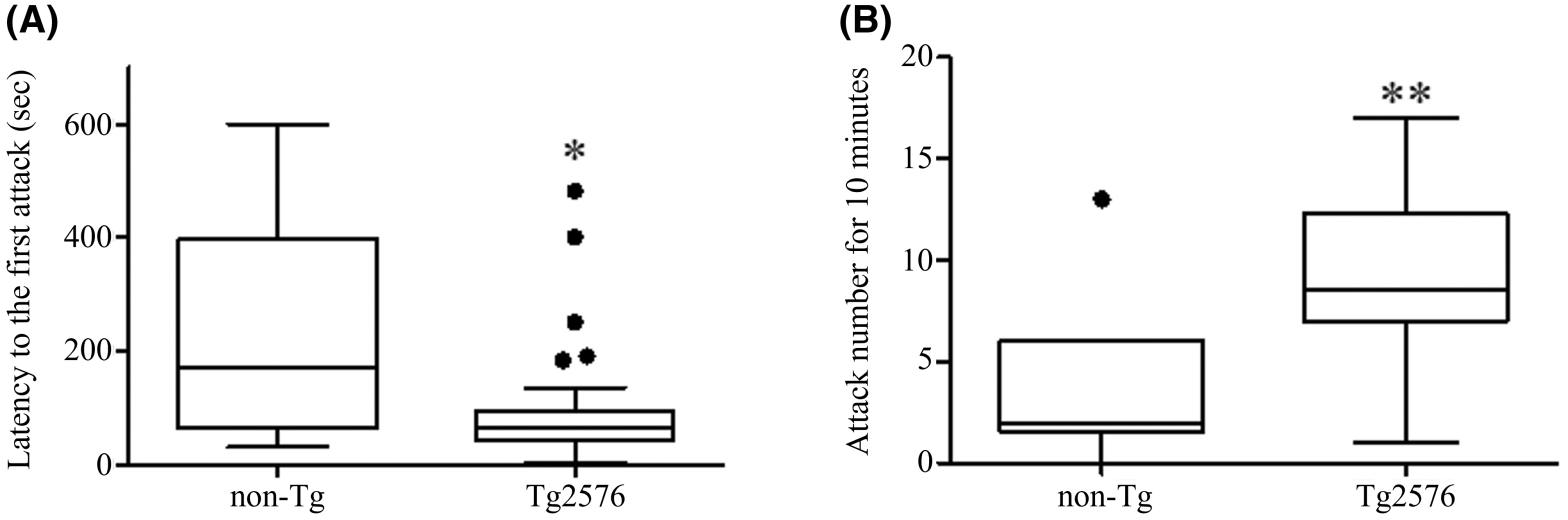
Increase of aggressive behavior in the Tg2576 mice in the resident–intruder test. (A) Latency time (second) to the first attack. The Tg2576 resident mice (*n* = 43) showed significantly shorter latency time to the first attack compared to the non‐Tg resident mice (*n* = 10). Most Tg2576 resident mice initiated biting intruder mice within the first 180 s of the test. Statistical analysis using the Wilcoxon rank‐sum test showed a significant reduction in the latency to the first attack in Tg2576 mice compared to non‐Tg mice (*p < 0.05). (B) Attack (biting) number for 10 min. Tg2576 resident mice exhibited significantly higher number of attacks than non‐Tg resident mice during the 10‐min test. Statistical analysis using the Wilcoxon rank‐sum test showed a significant increase in the number of biting in Tg2576 mice (**p < 0.01). Each rectangular box shows the interquartile range (25% and 75% percentiles) with the median within the box. The highest and lowest bars are the maximum and minimum values, respectively. The circle outside the box represents the outlier value.

Previously, Alexander et al.^15^ had reported the induction of aggression in Tg2576 mice using a resident–intruder test. Therefore, in the present study, both Tg2576 and non‐Tg mice were subjected to the same resident–intruder test at the age of 5 to 6 months. A total of 48 Tg2576 mice and 10 non‐Tg mice were included in the evaluation, with 5 Tg2576 mice excluded from the test due to their lack of aggressiveness. The results showed a significant reduction in time to first biting compared to the non‐Tg group (Figure 1A, *p* < 0.05, Wilcoxon rank‐sum test). The total number of biting over 10 min was also analyzed. As a result, the Tg2576 mice showed a significant increase in the number of biting (Figure 1B, *p* < 0.01, Wilcoxon rank‐sum test). In this way, the aggression suppressing effect of brexpiprazole was continuously investigated in the same Tg2576 mice, showing a clear promotion of aggressive behavior.

### 3.2 Brexpiprazole treatment of aggressive Tg2576 mice

Before conducting evaluation of brexpiprazole in Tg2576 mice, nonsedative doses of brexpiprazole were determined in B6SJLF1 mice in our in‐house study. After administration of brexpiprazole, spontaneous activity was measured for 1 h and statistically analyzed by the Williams test (two tailed). The doses at 0.01 and 0.03 mg/kg showed no significant differences compared to the vehicle control (data not shown). Therefore, these doses were selected for use.

**FIGURE 2:**
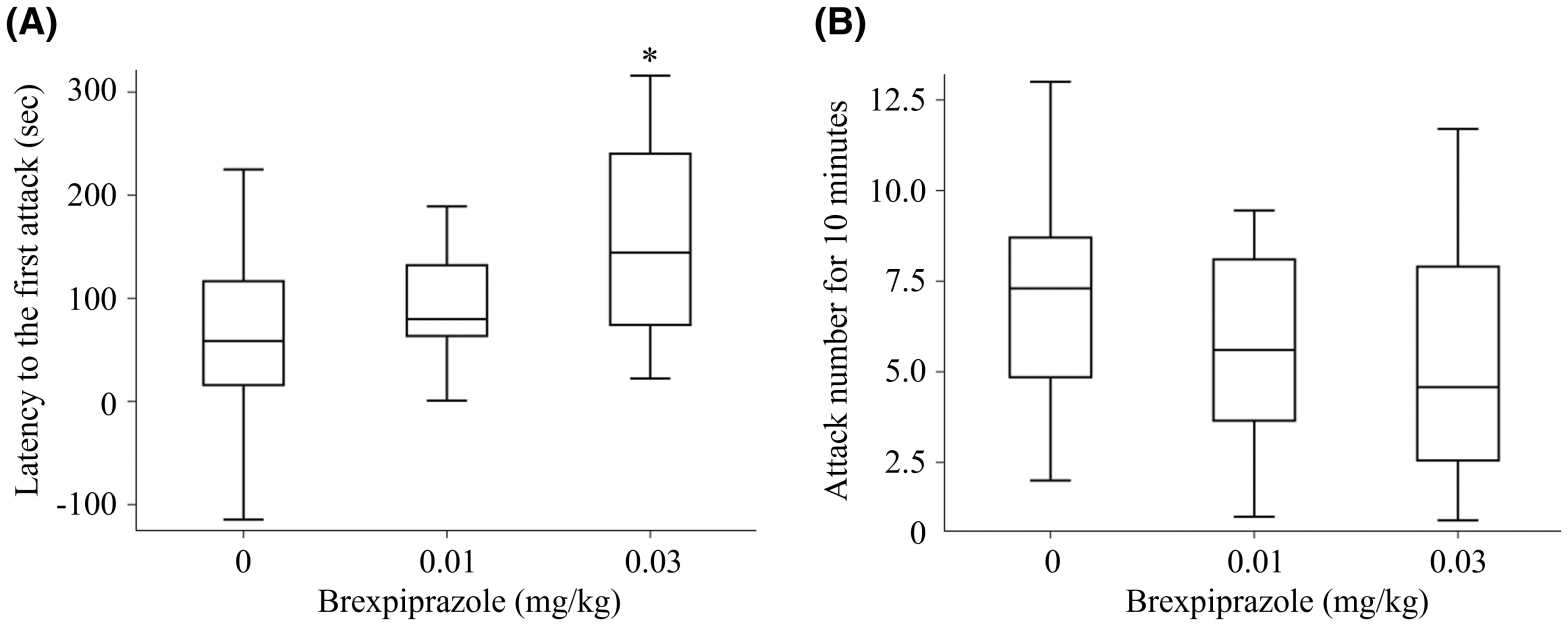
Suppressive effect of brexpiprazole on aggressive behavior in Tg2576 mice. (A) Latency time (second) to the first attack. The differences in latency to first attack between the vehicle (*n* = 15) and brexpiprazole (0.01 mg/kg, *n* = 14; 0.03 mg/kg, *n* = 14) groups were analyzed using ANCOVA (*p < 0.05), adjusted with baseline values of individual resident mice before treatment. (B) Attack (biting) number for 10 min. The differences in the number of attacks between the vehicle‐treated (*n* = 15) and brexpiprazole‐treated (0.01 mg/kg, *n* = 14; 0.03 mg/kg, *n* = 14) groups were analyzed using ANCOVA, adjusted with baseline values of individual resident mice before treatment. Data are presented as box plots showing medians (lines in boxes), 25% and 75% percentiles (boxes), and maximum and minimum values (whiskers).

Brexpiprazole was administered to Tg2576 mice at doses of 0.01 and 0.03 mg/kg 1 h before the start of the resident intruder test, and the aggression suppressing effect of brexpiprazole was investigated. The time to first biting was significantly prolonged in the 0.03 mg/kg group compared to the vehicle group (Figure 2A; *p* < 0.05, analyzed by ANCOVA). Besides, Tg2576 mice treated with 0.03 mg/kg brexpiprazole showed less biting number, albeit not significant, than mice treated with vehicle (Figure 2B).

### 3.3 Longitudinal assessment of circadian locomotor activity in APPSL‐Tg mice

**FIGURE 3:**
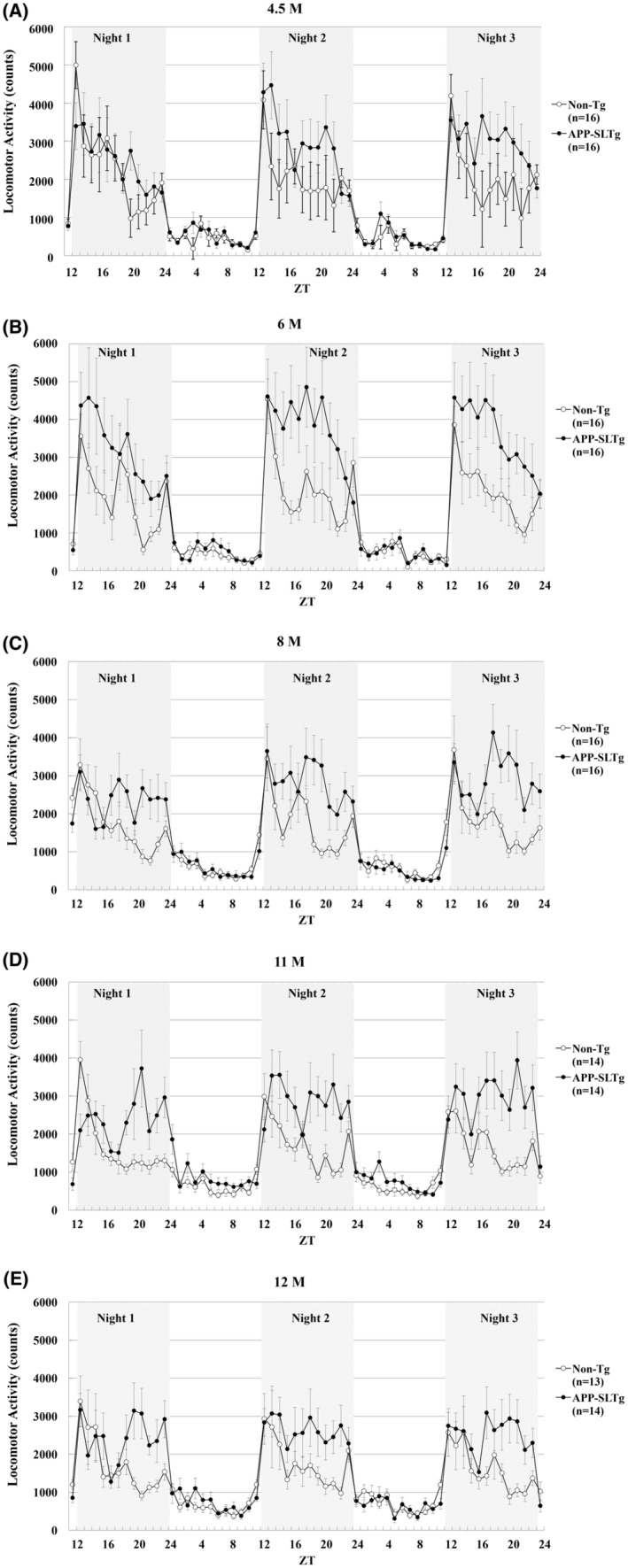
Infrared sensor‐based assessment of locomotion during light and dark periods from Night 1 to Night 3 in APPSL‐Tg and non‐Tg mice. The average hourly locomotor activity of each group was calculated and analyzed separately at each monthly (M) age (A: 4.5 M, B: 6 M, C: 8 M, D: 11 M, and E: 12 M). Zeitgeber time, ZT 0–12: light period, ZT 12–24: dark period (shaded). Data are expressed as mean ± SEM.

It is known that mice of the C57BL/6J strain, the background for APPSL‐Tg mice, show a clear distinction between light and dark periods, with a sharp increase in activity in the first hour of the dark period. In addition, the C57BL/6J strain shows a decrease in activity during the second part of the night. In preliminary experiments, APPSL‐Tg and non‐Tg mice were assessed in the locomotor activity study at different ages (4.5, 6, 8, 11, and 12 months) during the dark period (ZT12‐24) from Night 1 to 3 (Figure 3A–E, respectively). Total locomotor activity was significantly increased in APPSL‐Tg mice compared to non‐Tg mice during the dark period, and this increase occurred from 6 months of age (Figure 3B; Night 2: APPSL‐Tg mice, 45 402 ± 7819 counts, *n* = 16, non‐Tg mice, 26 546 ± 4519 counts, *n* = 16; Night 3: APPSL‐Tg mice, 42 793 ± 6836 counts, *n* = 16; non‐Tg mice, 25 137 ± 4031 counts, *n* = 16; *p* < 0.05 by unpaired *t*‐test, respectively). Furthermore, the significant increase in locomotor activity was observed up to 12 months of age when the longitudinal assessment was carried out (Figure 3E).

**FIGURE 4:**
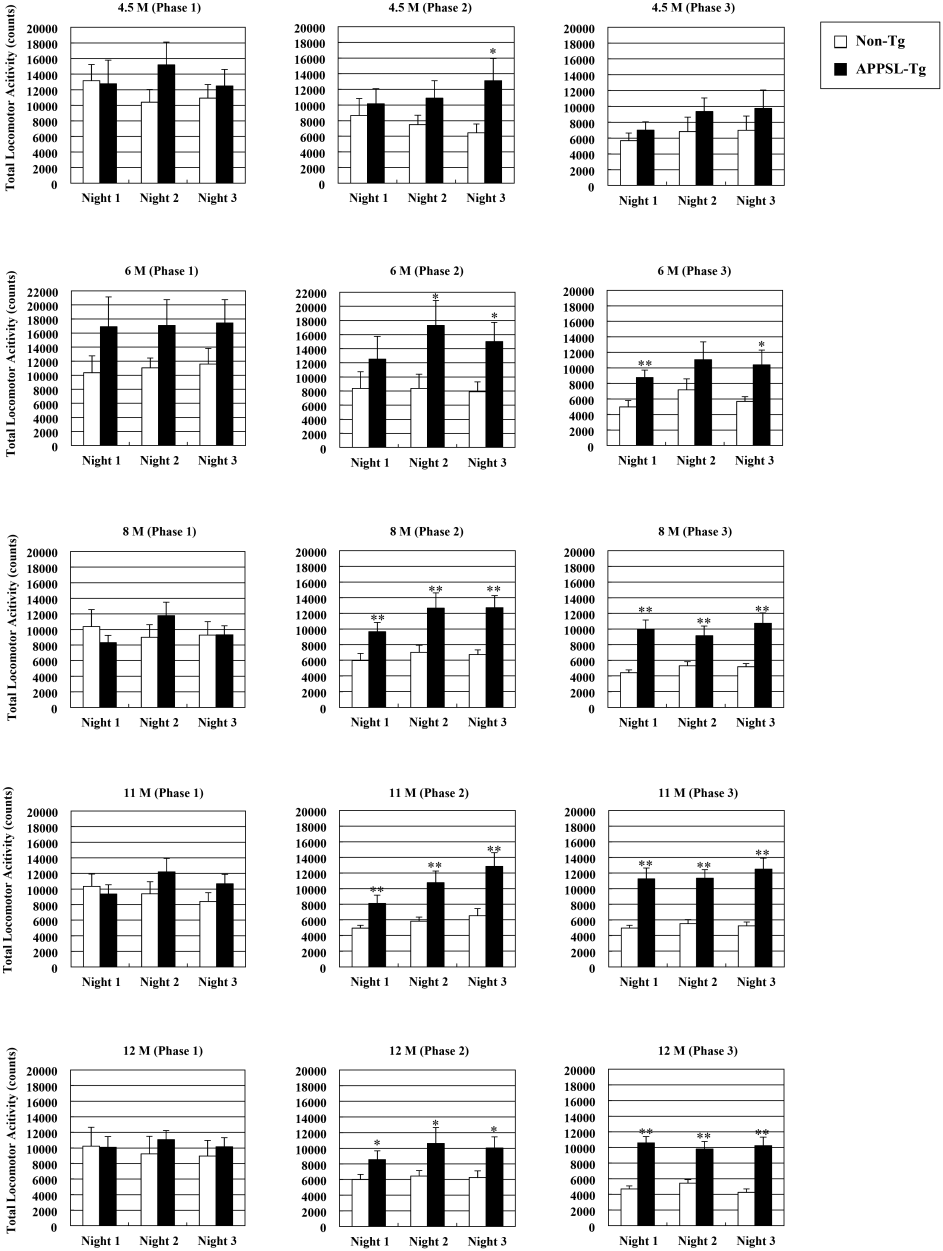
Longitudinal comparison of total locomotor activity per phase (I to III) in APPSL‐Tg and non‐Tg mice. The total locomotor activity of Phase I (ZT12‐16), Phase II (ZT16‐20), and Phase III (ZT20‐24) in dark period from Night 1 to 3 of APPSL‐Tg and non‐Tg mice was compared. *p < 0.05, **p < 0.01 versus non‐Tg mice, analyzed by the unpaired *t*‐test. Data are expressed as mean ± SEM.

When APPSL‐Tg and non‐Tg mice were compared by phase (I: ZT12‐16, II: ZT16‐20, and III: ZT20‐24), locomotor activities in Phases II and III were significantly increased in APPSL‐Tg mice from 6 to 12 months of age (*p* < 0.05 and *p* < 0.01 by unpaired *t*‐test, respectively, Figure 4). Based on this preliminary data, it was concluded that from 6 months of age, APPSL‐Tg and non‐Tg would be applicable for behavioral pharmacology experiments, and mice at this age were employed in subsequent brexpiprazole dosing studies.

Furthermore, as mentioned in Section 3.2, doses of brexpiprazole that did not affect nocturnal locomotion were determined in a preliminary dose finding study using non‐Tg mice aged 6 to 8 months (data not shown). Brexpiprazole at 0.01 and 0.03 mg/kg did not affect nocturnal locomotion and these doses were selected for investigation of the inhibitory effect of brexpiprazole on nocturnal hyperlocomotion in APPSL‐Tg mice.

### 3.4 Inhibitory effect of brexpiprazole on the nocturnal hyperlocomotion in APPSL‐Tg mice

Nocturnal locomotor activity was significantly increased in the APPSL‐Tg mice compared to non‐Tg mice in Phases II and III of the dark period. This increase was most prominently seen in Phase III in which the locomotor activity was significantly higher in APPSL‐Tg mice than non‐Tg mice through Night 1 to 3 (Table 1).

**TABLE 1:** Nocturnal locomotor activity of non‐Tg mice and APPSL‐Tg mice.

Dark period phase	Non‐Tg/vehicle (*n* = 5)			APPSL‐Tg/vehicle (*n* = 5)			*p*‐value
	Night 1	Night 2	Night 3	Night 1	Night 2	Night 3	
I	6204 ± 2596	6489 ± 2309	7260 ± 1524	9377 ± 2395	11 459 ± 3053	12 839 ± 1960	0.1804
II	5155 ± 1561	4678 ± 1199	3398 ± 518	8162 ± 1277	9438 ± 2146	13 249 ± 3101*	0.0250
III	2534 ± 597	2513 ± 765	3146 ± 616	9186 ± 955**	7469 ± 572**	10 694 ± 1329**	0.0001

*Note*: Locomotor activities of 6‐month‐old non‐Tg mice and APPSL‐Tg mice during dark period Phases I, II, and III through Night 1, 2, and 3 are shown. Both animals were treated with vehicle.

Data are expressed as mean ± SEM.

*p*‐values, shown in the table, are results analyzed by a MMRM method followed by Dunnett's test. *p < 0.05, **p < 0.01 versus non‐Tg mice, analyzed by an unpaired *t*‐test.

Therefore, the effect of brexpiprazole was evaluated in Phase III through Night 1 to 3. Nocturnal locomotor activity over time in Phase III of Night 1 to 3 was significantly suppressed in the brexpiprazole‐treated groups compared to the vehicle group (0.01 mg/kg group: *p* < 0.05; 0.03 mg/kg group: *p* < 0.05; Table 2). Additionally, brexpiprazole at 0.01 mg/kg significantly suppressed nocturnal hyperlocomotion activity in Phase III of Night 3 (*p* < 0.01; Table 2). Brexpiprazole at 0.03 mg/kg also suppressed locomotor activity of Tg‐mice in Phase III of Night 3 (*p* < 0.05; Table 2).

**TABLE 2:** Suppressive effect of brexpiprazole on nocturnal hyperlocomotion in APPSL‐Tg mice.

Dark period phase	APPSL‐Tg/vehicle (*n* = 5)			APPSL‐Tg/brexpiprazole 0.01 mg/kg (*n* = 6)			APPSL‐Tg/brexpiprazole 0.03 mg/kg (*n* = 5)		
	Night 1	Night 2	Night 3	Night 1	Night 2	Night 3	Night 1	Night 2	Night 3
III	9186 ± 1074	7469 ± 1074	10 694 ± 1074	7256 ± 980	6466 ± 980	4928 ± 980**	6014 ± 1074	5443 ± 1074	6845 ± 1074*
*p*‐value	—			0.045			0.046		

*Note*: Locomotor activities of 6‐month‐old APPSL‐Tg mice treated with vehicle or brexpiprazole (0.01 or 0.03 mg/kg) during dark period Phase III through Night 1, 2, and 3 are shown.

Data are expressed as least square mean ± SEM.

*p*‐values, shown in the table, are results analyzed by a MMRM method followed by Dunnett's test. **p < 0.01, *p < 0.05 versus APPSL‐Tg/vehicle, analyzed by a Dunnett's test.

## 4 DISCUSSION

Brexpiprazole was approved in United States for the treatment of agitation associated with dementia due to Alzheimer's disease (agitation in Alzheimer's dementia: AAD) in 2023.^12^, ^27^ In its clinical trials, efficacy, safety, and tolerability of brexpiprazole were demonstrated in patients with AAD.^11^, ^12^ Brexpiprazole is also approved as a treatment for schizophrenia and as an adjunctive therapy for MDD.^9^, ^28^, ^29^, ^30^, ^31^, ^32^ In association with these indications, effects of brexpiprazole have been demonstrated in animal models related to schizophrenia and MDD.^8^, ^33^, ^34^, ^35^ In this study, for confirmation of effects of brexpiprazole on BPSD‐like symptoms in animal models of Alzheimer's dementia, we evaluated the effects of brexpiprazole on aggressive behavior caused by resident–intruder test in Tg2576 mice and on increased nocturnal activity in APPSL‐Tg mice overexpressing a mutant form of APP with the Swedish and London mutations.

Physical aggression and agitation are seen in Alzheimer's dementia patients with high prevalence.^15^ In the resident–intruder test in our study, compared to non‐Tg mice, Tg2576 mice showed significantly higher attack number and shorter latency time to first attack, indicative of increased aggressiveness in the Tg mice. Our result is consistent with a previous report with Tg2576 mice by other researchers.^15^ We demonstrated that brexpiprazole (0.03 mg/kg) significantly delayed the latency to first attack and exhibited a trend toward a decrease in attack number in the Tg2576 mice.

APPSL‐Tg mice showed significantly higher nocturnal locomotor activity compared to non‐Tg mice most prominently during the dark period Phase III. Therefore, the effect of brexpiprazole was evaluated in the dark period Phase III (3:00 a.m.–7:00 a.m. [ZT 20–24]). Brexpiprazole (0.01 and 0.03 mg/kg) significantly decreased the nocturnal hyperlocomotion in APPSL‐Tg mice during the dark period Phase III. Similar to our results, Vloeberghs et al. reported that their Tg mice (APP23 mice) displayed nocturnal hyperlocomotion at 6 and 12 months old, especially during the second half of dark period, which they referred as reminiscent of sundowning syndrome in patients with Alzheimer's dementia.^22^ Although sundowning syndrome is still not understood well, it is a clinical phenomenon characterized by agitation, aggression, and delirium seen in patients with Alzheimer's dementia during the early evening hours.^36^

Taken together, these suggest that the suppression of attack behavior in brexpiprazole‐treated Tg2576 mice and the reduction of nocturnal hyperlocomotion in brexpiprazole‐treated APPSL‐Tg mice in our study are indicative of the therapeutic effect of brexpiprazole on AAD that was already confirmed in its clinical trials.

**FIGURE 5:**
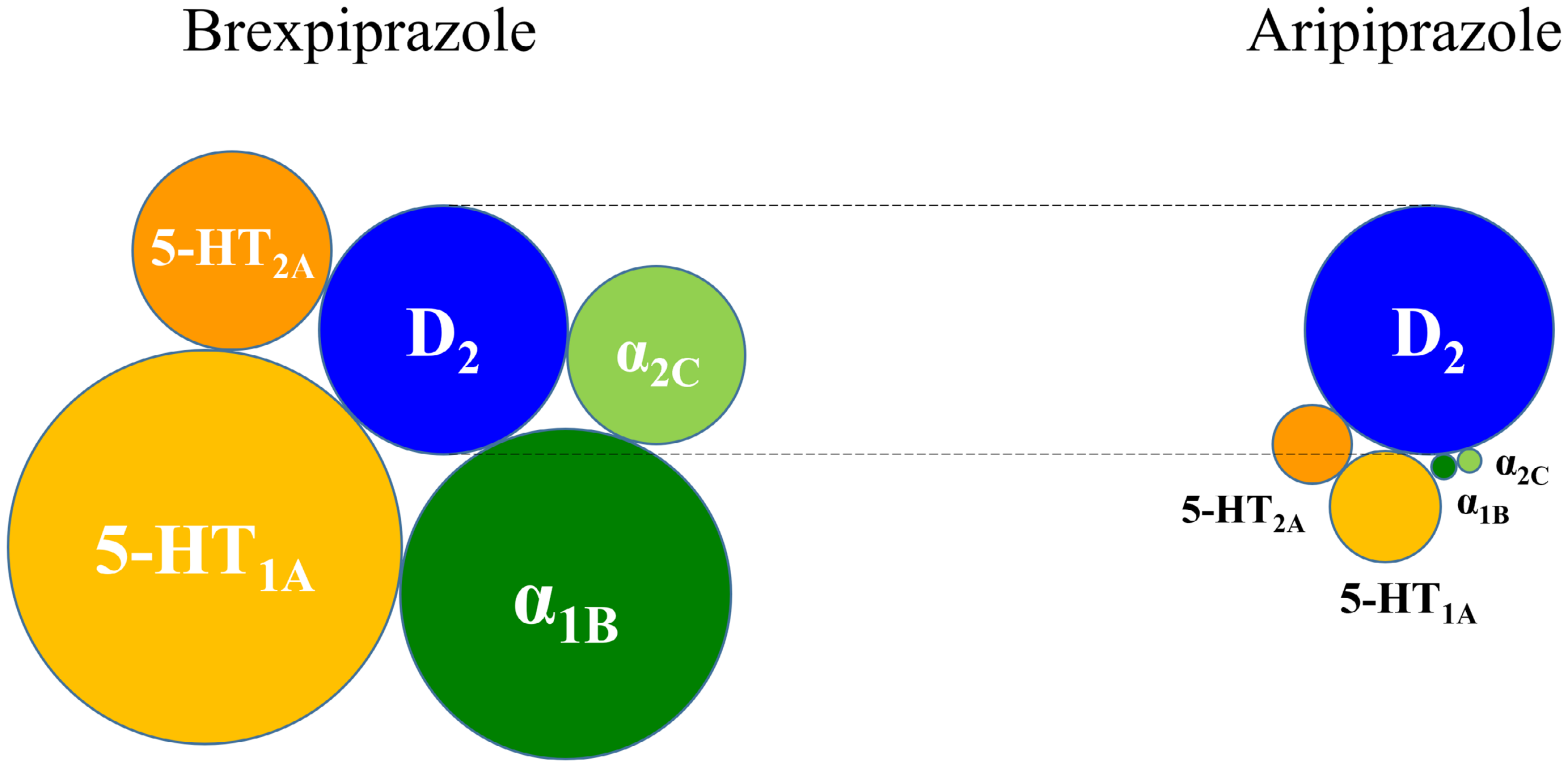
Comparative binding affinity of brexpiprazole to the human serotonin 5‐HT_1A_ and 5‐HT_2A_ receptors, and the human norepinephrine α_1B_ and α_2C_ receptors based on its affinity to the human dopamine D_2_ receptor. The strengths of binding affinity of brexpiprazole to the human serotonin 5‐HT_1A_ and 5‐HT_2A_ receptors, to the human norepinephrine α_1B_ and α_2C_ receptors, in comparison with that to the human D_2_ receptor were depicted based on its Ki values of the receptors.^8^ Those of aripiprazole were also depicted based on the Ki values of aripiprazole.

Although via what mechanism of action brexpiprazole suppressed attack behavior and nocturnal hyperlocomotion in these Tg mice and elicits therapeutic effects in AAD patients is currently unclear, brexpiprazole is known to act as a partial agonist at serotonin 5‐HT_1A_ and dopamine D_2_ receptors, and an antagonist at serotonin 5‐HT_2A_ and adrenalin α_1B_/α_2C_ receptors.^8^ Different from aripiprazole, brexpiprazole shows comparatively similar strengths of binding affinity to these receptors (Figure 5).

Association of 5‐HT_2A_ receptor polymorphisms with neuropsychiatric symptoms (i.e., BPSD) in Alzheimer's dementia has been reported.^37^, ^38^, ^39^ Although how the 5‐HT_2A_ receptor is involved in AAD is still unclear, DOI, a 5‐HT_2A_ receptor agonist, has been known to increase social isolation‐induced aggressive behavior in mice, which was inhibited by ritanserin, a 5‐HT_2A_ receptor antagonist.^40^ Not only relation of the 5‐HT_2A_ receptor but also of the 5‐HT_1A_ receptor to aggressive behavior has been reported. MKC‐242, a 5‐HT_1A_ receptor agonist, inhibited the DOI‐enhanced aggressive behavior.^40^ Moreover, buspirone, a 5‐HT_1A_ receptor agonist, attenuated social isolation‐induced aggressive behaviors in rats.^41^ It has also been reported that injection of 8‐OH‐DPAT, a 5‐HT_1A_ receptor agonist, into the ventral orbitofrontal cortex reduced the frequency of biting in the resident–intruder test in mice.^42^ This effect was reversed by injection of a 5‐HT_1A_ antagonist, WAY‐100635, into the same brain area.

The adrenergic α_2_ receptor density was increased in cortical membranes and dentate gyrus granule cell layer of post‐mortem brains of patients with Alzheimer's dementia,^43^, ^44^, ^45^ albeit α_2C_ adrenoceptor mRNA was decreased in the dorsal hippocampus.^45^ The α_2_ adrenoceptor was increased also in brain microvessels, innervated by locus coeruleus noradrenergic neurons, in patients with Alzheimer's dementia.^43^, ^46^ In addition, the α_2_ adrenoceptor levels in cerebellar cortex were increased in agitated aggressive Alzheimer's dementia patients compared with nonagitated Alzheimer's dementia patients.^47^ The α_2A_ and α_2C_ adrenoceptors are the primary α_2_ adrenoceptor subtypes working in the CNS. It has been recognized that the α_2C_ adrenoceptor plays a distinct and specific role in memory, cognition, and mood disorders in a different manner from the role of the α_2A_ adrenoceptor.^48^ The α_2C_ adrenoceptor antagonism improves spatial memory, working memory, and prepulse inhibition deficit.^48^ Although mRNA levels and protein activity of the α_1B_ adrenoceptor were almost the same between Alzheimer's dementia and normal brains, mRNA and protein levels of tissue transglutaminase (TGII), α_1B_ adrenoceptor signal mediator, were increased in Alzheimer's dementia brains.^49^ The α_1B_ adrenoceptor is suggested to play a role in fear‐motivated exploratory behavior.^43^ Interestingly, prazosin, an α_1_ adrenoceptor antagonist, improved behavioral symptoms in Alzheimer's dementia patients with agitation and aggression, although a further verification is needed as the study size was small.^50^ Prazosin nonselectively binds to α_1A_ and α_1B_ adrenoceptors.^51^ Thus, it is unclear which antagonist activity at α_1A_ or α_1B_ adrenoceptors is responsible for prazosin's ameliorating effect. However, considering that brexpiprazole has selectivity to the α_1B_ adrenoceptor, the antagonist activity at the α_1B_ adrenoceptor may be responsible for ameliorating effect on AAD.

It has been reported that striatal D_2_/D_3_ receptor availability was increased in Alzheimer's dementia patients with delusions.^52^ Ventral tegmental area (VTA) dopaminergic neurons projecting to the lateral septum has been reported to be sufficient for promoting aggressive behavior in mice, in which D_2_ receptor signaling is necessary for VTA dopaminergic activity to promote aggression.^53^ In contrast, 4‐week isolated mice increased aggressive behavior and D_2_ receptor density was reduced in medial prefrontal cortex and nucleus accumbens.^54^ D_2_ receptor agonist quinpirole ameliorated these changes. These suggest that D_2_ receptor partial agonists, by stabilizing dopamine signals, may be an appropriate option for treatment of aggressive behavior compared to merely D_2_ receptor full antagonists or agonists.

Therefore, serotonin 5‐HT_1A_ receptors, 5‐HT_2A_ receptors, adrenoceptor α_1B_, α_2C_, and dopamine D_2_ receptors are implicated in the pathology of BPSD in Alzheimer's dementia, and brexpiprazole's partial agonist or antagonist activities at these receptors may be responsible for its ameliorating effect on agitation in patients with Alzheimer's dementia. Moreover, the balance of brexpiprazole's partial agonist or antagonist activities at these receptors may also support the tolerability observed in clinical trials of patients with AAD.^11^, ^12^

## 5 CONCLUSIONS

The suppression of attack behavior in brexpiprazole‐treated Tg2576 mice and the reduction of nocturnal hyperlocomotion in brexpiprazole‐treated APPSL‐Tg mice in our study support the therapeutic effect of brexpiprazole on AAD as demonstrated in the clinical trials.

## CONFLICT OF INTEREST STATEMENT

All the authors are employees of Otsuka Pharmaceutical Co., Ltd. NA, SS, DI, MS, TF, and KM own stock in Otsuka Holdings Ltd. Additionally, SS, DI, MN, and KM hold a patent (WO2014/065437) related to brexpiprazole. The authors affirm that these potential conflicts of interest have been duly acknowledged and addressed in compliance with the journal's policy.

## ETHICAL STATEMENT

Approval of the Research Protocol by an Institutional Reviewer Board: The experimental procedure in this study was approved and conducted with Guidelines for Animal Care and Use in Otsuka Pharmaceutical Co., Ltd.

Informed Consent: N/A.

Registry and the Registration No. of the Study/trial: N/A.

Animal Studies: The care and handling of the animals was in accordance with “Guidelines for Animal Care and Use in Otsuka Pharmaceutical Co, Ltd.”

## Supporting information


Data S1.

## Data Availability

All data are available in the Supporting Information data file (Data S1).
