# Correction to “Brexpiprazole: A new option in treating agitation in Alzheimer's dementia‐Insights from transgenic mouse models”

**DOI:** 10.1002/npr2.12496

**Published:** 2024-11-12

Amada N, Sato S, Ishikawa D, Nakamura M, Suzuki M, Futamura T, Maeda K. Brexpiprazole: a new option in treating agitation in Alzheimer's dementia‐Insights from transgenic mouse models. Neuropsychopharmacol Rep 2024. doi: https://doi.org/10.1002/npr2.12461.

In paragraph 4 of the “Introduction” section, the last sentence includes the text “Swedish and London‐type amyloid precursor protein (APP) mutations”. This is incorrect.

It should be: Swedish‐type amyloid precursor protein (APP) mutation.

In the first paragraph of the sub‐section “2.3. Resident‐intruder test”, the eighth sentence includes the text “A/J mice did not show any nonaggressive behavior”. This is incorrect.

It should be: A/J mice did not show any aggressive behavior.

We apologize for these errors.

